# Correction: Toxicity of metal–organic framework nanoparticles: from essential analyses to potential applications

**DOI:** 10.1039/d3cs90014b

**Published:** 2023-01-19

**Authors:** Romy Ettlinger, Ulrich Lächelt, Ruxandra Gref, Patricia Horcajada, Twan Lammers, Christian Serre, Patrick Couvreur, Russell E. Morris, Stefan Wuttke

**Affiliations:** a School of Chemistry, University of St. Andrews St. Andrews UK rle6@st-andrews.ac.uk; b Department of Pharmacy and Center for NanoScience (CeNS), Ludwig-Maximilians-Universität München Munich Germany; c Division of Pharmaceutical Technology and Biopharmaceutics, University of Vienna Vienna Austria; d Institut de Sciences Moléculaires d’Orsay, Université Paris Saclay Paris France; e Madrid Institute for Advanced Studies Madrid Spain; f Institute for Experimental Molecular Imaging, RWTH Aachen University Clinic Aachen Germany; g Département de Chimie, Ecole Normale Supérieure de Paris Paris France; h Institut Galien Paris-Sud, Université Paris Saclay Paris France; i Ikerbasque, Basque Foundation for Science Bilbao Spain; j Basque Center for Materials, UPV/EHU Science Park Leioa Spain stefan.wuttke@bcmaterials.net

## Abstract

Correction for ‘Toxicity of metal–organic framework nanoparticles: from essential analyses to potential applications’ by Romy Ettlinger *et al.*, *Chem. Soc. Rev.*, 2022, **51**, 464–484, https://doi.org/10.1039/D1CS00918D.

The authors regret that the information presented in Fig. 12 and discussed in the text on p. 478 may have been unclear, or inconsistent with the information derived from ref. 26. The corrected version of Fig. 12 is presented here. The corresponding sentence on p. 478, beginning, “Herein, endogenous fumaric acid…”, should also be revised as follows:

“Herein, nitro- and tetramethyl-terephthalic acid modifications turned out to be the most biofriendly organic linkers, followed by exogenous trimesic acid, amino-terephthalic acid, and terephthalic acid, and the endogenous fumaric acid had the lowest assessed IC_50_ value.”

**Fig. 12:**
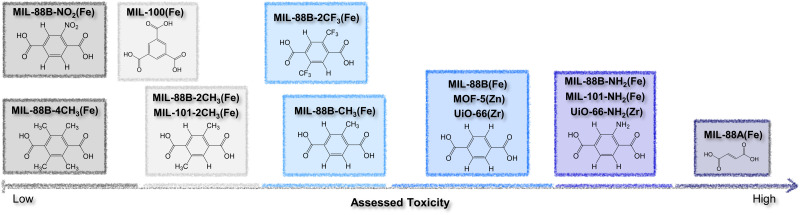
Ranking of different organic linkers, based on their reported IC_50_ toxicity data.

The Royal Society of Chemistry apologises for these errors and any consequent inconvenience to authors and readers.

## Supplementary Material

